# Metabotropic glutamate receptor 5 binding in male patients with alcohol use disorder

**DOI:** 10.1038/s41398-017-0066-6

**Published:** 2018-01-10

**Authors:** Funda Akkus, Yoan Mihov, Valerie Treyer, Simon M. Ametamey, Anass Johayem, Smeralda Senn, Susanne Rösner, Alfred Buck, Gregor Hasler

**Affiliations:** 1 Division of Molecular Psychiatry, Translational Research Center, University Hospital of Psychiatry, University of Bern, 3000 Bern 60, Switzerland; 2 PET Center, Division of Nuclear Medicine, University Hospital, 8091 Zurich, Switzerland; 3 Center for Radiopharmaceutical Science of ETH, PSI, and USZ, Department of Chemistry and Applied Biosciences of ETH, 8093 Zurich, Switzerland; 4 Forel Clinic, Addiction Treatment Center, 8548 Ellikon an der Thur, Switzerland

## Abstract

Glutamate signaling plays a major role in addiction. Preclinical research strongly suggests an implication of G-protein-coupled metabotropic glutamate receptor subtype 5 (mGluR5) in nicotine addiction and alcohol use disorder. In humans, smoking is related to a global reduction in mGluR5 availability. In the present study, we investigated mGluR5 in vivo in patients with alcohol use disorder without the confounding effects of smoking. A total of 14 male subjects with alcohol use disorder and at least a 25-day abstinence and 14 matched male non-smoking healthy controls were included in the study. We employed positron emission tomography (PET) with the mGluR5-specific radiotracer [11C]ABP688, using a bolus/infusion protocol. We found increased mGluR5 DVR in several regions within the temporal lobe in patients, as compared to controls. The largest between-group difference was in the amygdala. There was a marked positive relation between mGluR5 DVR in the anterior cingulate and mGluR5 DVR in the orbitofrontal cortex in patients, but not in controls. In patients, lower temptation to drink was related to higher amygdala mGluR5 DVR. We did not find altered mGluR5 DVR in the basal ganglia of subjects recovering from alcohol use disorder. In conclusion, our study provides clinical evidence for altered mGluR5 signaling in the amygdala in alcohol use disorder. This alteration was associated with the temptation to drink. In addition, this study suggests abnormal mGluR5 signaling in a network underlying reward-related behavioral flexibility. These findings strengthen the case for pharmacological agents acting on mGluR5 as promising candidates for the treatment of alcohol use disorder.

## Introduction

Alcoholism is an important risk factor for many disease conditions, including neuropsychiatric disorders, and a major contributor to years of life lost to disability and premature death.^1^ Alcohol use disorder is characterized by intense craving and compulsive intake of alcohol despite its adverse consequences.^2^ Glutamate signaling in the brain plays a major role in the pathogenesis of addiction disorders.^3–5^ Glutamate acts on ionotropic receptors and G-protein-coupled metabotropic receptors, of which, currently, eight subtypes with distinct molecular and pharmacological properties have been described (metabotropic glutamate receptor subtypes 1–8 (mGluR1–8), correspondingly).^6^ Preclinical studies implicate mGluR5 in binge alcohol intake.^7–9^ Prolonged alcohol intake can increase mGluR5 protein levels in the nucleus accumbens and the amygdala, depending on the experimental protocol.^10–13^ Reversely, systemic administration of mGluR5 negative allosteric modulators (NAMs) can inhibit alcohol self-administration.^7^ More specifically, intracranial injections of mGluR5 NAMs directly into the nucleus accumbens and the amygdala reduce alcohol intake and reinstatement of alcohol seeking.^11–16^ Altogether, these preclinical studies provide a strong rationale for investigating mGluR5 in the amygdala and the nucleus accumbens in patients with alcohol use disorder.

A recent study employing positron emission tomography (PET) with the mGluR5 tracer ^18^F-3-fluoro-5-[(pyridin-3-yl)ethynyl] benzonitrile (^18^F-FPEB) addressed this issue.^17^ However, 11 out of the 16 patients in that study were smokers, whereas all controls were non-smokers. Our work has demonstrated a global reduction in mGluR5 in current smokers and ex-smokers.^18,19^ Therefore, a study focusing on non-smoking participants is needed to exclude the confounding effects of smoking on mGluR5. To this end, we investigated mGluR5 in non-smoking male patients recovering from alcohol use disorder and healthy non-smoking male controls, employing PET with the mGluR5-specific radiotracer 3-(6-methyl-pyridin-2-ylethynyl)-cyclohex-2-enone-O-^11^C-methyl-oxime ([^11^C]ABP688).^20,21^ We compared mGluR5 binding between patients and controls throughout the brain, with a focus on the nucleus accumbens and the amygdala. Since alcohol use disorder displays aberrant resting-state connectivity in functional magnetic resonance imaging (fMRI) studies,^22,23^ we also investigated how correlations of mGluR5 binding among different brain regions differ in patients vs. controls.

## Materials and methods

### Participants

Study participants comprised non-smoking individuals who met the diagnostic criteria for moderate (*n* = 3) or severe (*n* = 11) alcohol use disorder with at least 25 (SD ± 18.1) days of continuous abstinence from admission to the hospital to the day of the scan, according to DSM-5 (Diagnostic and Statistical Manual of Mental Disorders, Fifth Edition; American Psychiatric Association, 2013), and healthy controls (*n* = 14). Only male patients were included in the study because we did not find female patients who fulfilled the inclusion criteria. We did not exclude research subjects from the analysis. Table 1 shows the demographic and clinical characteristics of the two samples. Patients were recruited through the Forel Hospital, Alcohol Relapse Prevention Unit, Ellikon an der Thur, Switzerland, and were all inpatients during study participation. Controls were recruited through online advertisements. We chose the sample size based on our previous PET studies with this tracer.^18,19,24,25^

**Table 1:** Demographic and clinical characteristics of the study samples

	Patients	Healthy controls
Age	46.6 (13.6)	44.1 (12.4)
Gender (male/female)	14/0	14/0
Onset alcohol use disorder		
Age >18 years	*n* = 7	—
Age >40 years	*n* = 7	—
Duration of alcohol use disorder, in years	9.4 (7.8)	—
BDI	3.5 (3.2)	1.1 (1.7)^a^
BAI	2.1 (1.9)	0.9 (1.9)
AUDIT^b^	25 (7.5)	—
Number of drinks per week prior to admission	77 (49)	4.2 (2.5), information from 6 participants only
Tobacco/cannabis consumption	—	—
Medication	*n* = 10, of these: 7 antidepressants (1 plus Ritalin), 1 Campral, 1 Lyrica, 1 Lyrica and Keppra	—
Highest education	High school: *n* = 9; college: *n* = 4; academic: *n* = 1	High school: *n* = 2; college: *n* = 8; academic: *n* = 4
Psychiatric history	MDD *n* = 8; one also has ADHD	—

Numeric values represent the mean (SD), unless indicated otherwise. *N* refers to the number of participants within this category

^a^*P*-value < 0.05, two-sided, comparing patients and controls with Welch′s test for independent samples

^b^*AUDIT*=Alcohol use disorders identification test (a score of ≥8 indicates harmful or hazardous drinking; score ≥15 in men indicates alcohol use disorder)

Participants were included into the study only after full explanation of the goals and procedures of the study and the risks of the study procedures. Informed consent was obtained from all subjects. The study and the written consent were approved by the local ethics committee (Kantonale Ethikkommission Zürich).

A psychiatrist and a psychologist carried out all interviews and clinical assessments. Psychiatric diagnoses were established via an unstructured clinical interview by the psychiatrist and a structured interview using the *Structured Clinical Interview for the DSM*.^2^ Exclusion criteria for patients comprised current medical or neurological disorder, abnormal blood coagulation, cigarette smoking, nuclear medicine examination in the last 5 years, major comorbid psychiatric conditions such as schizophrenia, bipolar disorder, current depressive episode and addiction disorders other than alcohol use disorder. Smoking and psychiatric and medical conditions were exclusion criteria for controls.

Additional clinical measures included the Beck Anxiety Inventory (BAI),^26^ the Beck Depression Inventory (BDI),^27^ the Alcohol Use Disorders Identification Test (AUDIT)^28^ and the Alcohol Abstinence Self-Efficacy Scale (AASE).^29^ Based on the AASE, craving for the rewarding effects of alcohol was calculated as the sum of items 4, 8, 15, 17 and 20 (reward craving), whereas craving for the stress relieving effects of alcohol was measured with the sum of items 3, 6, 12, 16 and 18 (relief craving), as previously suggested.^30^

In order to assess the relationship between antidepressant medication, mGluR5 distribution volume ratio (DVR) and relapse, we calculated imipramine dose equivalents for all antidepressants. These calculations were based on dose conversion factors proposed elsewhere.^31^

### Positron emission tomography

We used a bolus/infusion protocol evaluated in our previous work.^18,32^ A total of 570–624 MBq of [^11^C]ABP688 in a 50 ml volume was administered using an infusion pump (half was given as a bolus over 2 min and the other half was infused over the next 58 min). Image acquisition and reconstruction were performed as previously described.^18^ All scans started between 1530 and 1600 h local time. Visual inspection of the tissue time activity curves did not reveal differences between diagnostic groups with respect to tracer kinetics.

### Statistical analysis

We based our analyses on a maximum probability atlas implemented in the PMOD PNEURO-Tool (Version 3.6; PMOD Technologies, Switzerland, www.pmod.com). For participants, no MRIs were available. Therefore, the maximum probability atlas and automatic region of interest segmentation was based on the early frames of the PET which were normalized to the template. The template-based segmented regions were then automatically back projected on the original individual PET image. The regions of interest were visually controlled by a trained reader and slight corrections were applied if needed to ensure correct placement (especially in the basal ganglia area). The DVR for each region of interest was calculated based on the late imaging frames showing equilibrium (45–60 min) and the cerebellum as a reference region as previously reported for the bolus-infusion paradigm.^32–34^ Two-tailed Welch's tests, which do not assume equal variance in the compared samples, and General Linear Model repeated measures analyses were used to test the differences in mGluR5 DVR and clinical values between and within the groups. We did not estimate variance and did not test variance equality. We did not perform partial volume corrections as no MRI was available to assess and correct for alcohol use disorder-related atrophy. The standard maps were manually adapted for regions such as the amygdala and caudate in case the outline was not acceptable based on the early-phase image (perfusion like maps). Therefore, a correction for individual local size differences was performed to reduce potential effects of reduced gray matter thickness.

We assessed correlations between mGluR5 DVR among brain regions and inter-regional correlation differences between patients and controls in a three-step procedure. First, we calculated a correlation matrix comprising 36 brain regions within each group using Pearson's correlation coefficient. This resulted in 630 correlations per group reflecting the relation between mGluR5 DVR in 630 unique pairs of brain regions. In a second step, we compared both groups by applying an* r*-to*-z* transformation to all 630 unique correlations in each group and calculating Welch's test (package “psych” version 1.7.5 in R version 3.3.2, R Foundation for Statistical Computing, Vienna, Austria, available at https://cran.r-project.org/). Following this global between-group comparison, in a final step, we compared each one of the 630 unique correlations between controls and patients. We carried out these individual comparisons with Fisher′s *r*-to-*z* transformation, as implemented in the package “cocor” (version 1.1–3) in R. We set a significance threshold of *p* < 0.001 (one-tailed, uncorrected for multiple testing). For illustration purposes, we displayed the correlation matrices in both groups separately and the numerical correlation differences between patients and controls as color maps (Figures S1-S2 and Fig. 2a, respectively).

**Fig. 1:**
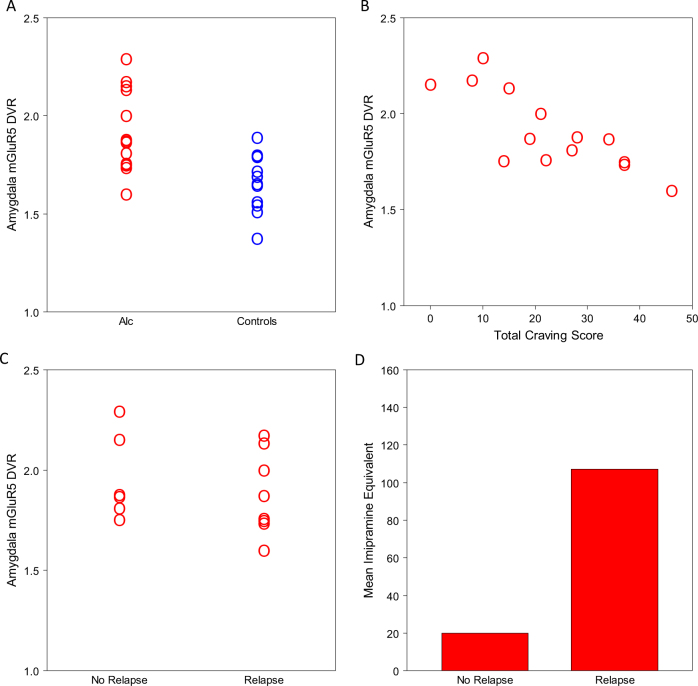
**a** Amygdala mGluR5 DVR in patients (red) and controls (blue). **b** The relation between mGluR5 DVR in the amygdala and the total craving score of the AASE in the patient group.^30^ **c** Amygdala mGluR5 DVR in patients who reported a relapse after 3–6 months and those who reported they remained abstinent. **d** The mean imipramine equivalent dose of antidepressants at the timepoint of scanning in patients who reported a relapse, as compared to those who remained abstinent

**Fig. 2:**
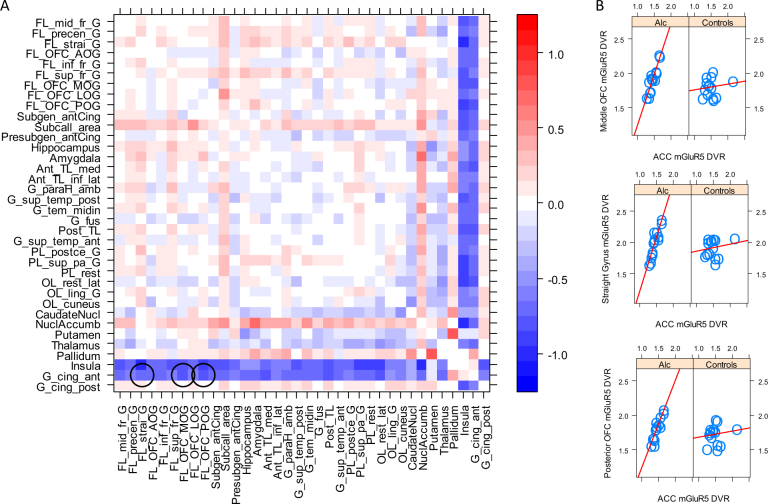
Inter-regional mGluR5 DVR correlations across various brain structures differ between healthy controls and patients with alcohol use disorder. **a** For each inter-regional correlation the numeric difference between healthy controls and patients is shown, calculated as *r*_controls_−*r*_patients_. Color heat represents the direction and magnitude of this difference, as indicated in the color bar. Thus, red squares indicate higher correlation in controls than in patients, whereas blue indicates higher correlation in patients than in controls. Black circles highlight the most pronounced differences in ACC–OFC and ACC–straight gyrus mGluR5 DVR correlations between patients and controls (*p* < 0.001, one-tailed, uncorrected for multiple comparisons). **b** Shown are the highlighted correlations in each group separately. The red line represents a linear regression estimate

We assumed that the data are normally distributed. Explorative analyses of mGluR5 DVR values with Shapiro–Wilk tests yielded significant deviations from normality in healthy controls in the straight gyrus, the posterior part of the superior temporal gyrus, the inferiolateral remainder of the parietal lobe, pallidum, insula and the anterior cingulate gyrus (*p* < 0.05, uncorrected for multiple comparisons). We also found significant deviations from normality in subjects with alcohol use disorder in the superior parietal gyrus and the cuneus (*p* < 0.05, uncorrected for multiple comparisons). Based on these results, we decided to display individual data points in scatterplots for between-group comparisons of mGluR5 DVR values and for between-group comparisons of correlations (Figs. 1 and 2) to demonstrate that our findings are not a statistical artifact caused by outliers.

## Results

Table 1 presents a summary of the demographic and clinical characteristics of all study participants. The gender of all participants was male. Age did not differ significantly between groups (*t*_(25.79)_ = 0.49, *p* = 0.63). Patients reported significantly higher BDI scores (*t*_(19.99)_ = 2.5, *p* = 0.02) and BAI scores that were higher but did not reach criteria for statistical significance (*t*_(26)_ = 1.7, *p* = 0.1).

To compare mGluR5 DVR between both groups, we carried out a multivariate analysis of covariance with mGluR5 DVR in 36 brain regions as dependent variables, group as a fixed factor and BDI and BAI as covariates. Whereas no multivariate difference between both groups was found (*p* > 0.05), post hoc analysis of variance (ANOVA) for all regions indicated that mGluR5 DVR was significantly increased in various brain regions in patients, mainly in the temporal lobe (Table 2). The most prominent effect was in the amygdala (F_(1, 26)_ = 11.76, *p* = 0.005; Fig. 1a).

**Table 2:** Comparison of mGluR5 DVR in subjects with alcohol use disorder and healthy controls

Brain region^a^	Patients mean (SD)	Controls mean (SD)	F_(1, 26)_	*p*-value	Partial *ε*^2^
Hippocampus	1.76 (0.18)	1.65 (0.12)	3.8	0.062	0.127
Amygdala	1.91 (0.20)	1.69 (0.14)	11.76	0.005	0.311
Anterior temporal lobe, medial part	1.94 (0.16)	1.80 (0.13)	6.24	0.019	0.194
Anterior temporal lobe, lateral part	2.00 (0.16)	1.84 (0.13)	9.38	0.005	0.265
Parahippocampal and ambient gyri	1.85 (0.20)	1.65 (0.13)	9.06	0.006	0.258
Superior temporal gyrus, posterior part	1.79 (0.13)	1.70 (0.12)	3.89	0.059	0.130
Middle and inferior temporal gyrus	1.92 (0.16)	1.78 (0.13)	6.13	0.020	0.191
Gyrus fusiformes	2.01 (0.19)	1.87 (0.12)	4.95	0.035	0.160
Posterior temporal lobe	1.79 (0.14)	1.68 (0.09)	6.06	0.021	0.189
Superior temporal gyrus, anterior part	1.83 (0.15)	1.70 (0.13)	5.56	0.026	0.176

^a^Brain regions as implemented in PMOD (http://doc.pmod.com/pneuro/7674.htm); mean (SD) refers to the average and the standard deviation of the mGluR5 DVR in the corresponding brain region in patients and controls; F-values for the effect of group in post hoc ANOVAs; *p*-values for the effect of group; partial *ε*^2^, as calculated in SPSS, indicates the effect size

Next, we investigated the relation between amygdala mGluR5 DVR and clinical variables. We found negative correlations between amygdala mGluR5 binding and the AASE scores for reward craving and relief craving (rho = −0.79, *p* = 0.001; rho = −0.8, *p* = 0.001, respectively, Fig. 1b). Age, alcohol consumption per week (glasses), duration of alcohol use disorder (years), age of onset and abstinence duration prior to scan did not correlate with mGluR5 DVR in the amygdala (all *p* > 0.07). For the other brain regions we found no significant correlations with any of these variables except for a negative correlation between mGluR5 DVR in the superior frontal gyrus (*r* = −0.593, *p* < 0.05) and superior parietal gyrus (*r* = −0.622, *p* < 0.05) with age. Furthermore, we found no significant difference in mGluR5 DVR between patients with and without family history of alcohol use disorder in any brain region or any of the clinical parameters mentioned above. Amygdala mGluR5 DVR was higher in medication-free persons (*n* = 4) with alcohol use disorder than those taking antidepressants (*n* = 7, *t*_(7.813)_ = 3.539, *p* < 0.01).

Patients with MDD in their history did not show different mGluR5 DVR in the amygdala or any other region compared to those without MDD in the past. Importantly, no patient fulfilled the diagnostic criteria for current MDD. Thus, the use of antidepressants was not related to an acute depressive disorder, but provided to prevent further depressive episodes in remitted patients or to reduce depressive symptoms during withdrawal from alcohol. In the seven cases treated with antidepressants, the mean imipramine equivalent dose was 139.4 ± 97.8 (average ± standard deviation). Within the patient sample (*n* = 14), imipramine equivalent correlated negatively with mGluR5 DVR in the subgenual anterior cingulate region (*r* = −0.56, *p* < 0.05; uncorrected for multiple comparisons).

After 3 to 6 months we contacted the patients by telephone to ask whether they relapsed. Out of 14 participants, 8 reported that they relapsed during this time window. We found no significant relation between relapse and amygdala mGluR5 DVR (Fig. 1c) or any other imaging or clinical parameter, except the use of antidepressants. Antidepressant medication at the time of scanning, as estimated by imipramine equivalents, was higher in patients who relapsed. However, this difference did not reach statistical significance (*t*_8.28_ = 2.02; *p* = 0.08, two-tailed; Fig. 1d).

The inter-regional correlation analysis showed a global pattern of positive correlations between mGluR5 DVR in various brain regions across the entire brain in both groups (Figures S1 and S2). Overall, correlations were higher in patients than in controls, as indicated by Welch's two-sample *t*-test of *z*-transformed correlation values (*t*_1189.8_ = 2.21, *p* = 0.03, two-tailed, Figure S3). Welch's two-sample *t*-test with the non-transformed correlation values yielded a similar result (*t*_1101.7_ = 3.53, *p* < 0.01, two-tailed). At a descriptive level, the graphical representation of the correlation differences between groups suggested that mGluR5 DVR in the anterior cingulate cortex (ACC) and the insula were more strongly related to mGluR5 DVR in other brain regions in patients than in controls (Fig. 2a). This difference was particularly strong for the correlations between mGluR5 DVR in the ACC and three anatomically contiguous structures, the straight gyrus, the middle and the posterior orbitofrontal cortex (OFC). Whereas in patients higher mGluR5 in the ACC closely corresponded to higher mGluR5 in the middle, the posterior OFC and the straight gyrus, we did not find such relations in controls (*p* < 0.001, one-tailed, uncorrected for multiple comparisons; Table S1, Fig. 2b). A simulation analysis with randomly generated values corroborated the validity of these results (see Supplementary Information, Figures S4 and S5).

## Discussion

The present study investigated mGluR5 DVR in male non-smoking patients with alcohol use disorder after abstinence for at least 25 days compared to healthy age-matched non-smoking male controls. We found increased mGluR5 DVR in various brain regions located in the temporal lobe in alcohol use disorder. This effect was particularly strong in the amygdala. In patients, but not in controls, higher mGluR5 DVR in the ACC corresponded to higher mGluR5 DVR in the medial, posterior OFC and the straight gyrus. In patients, lower temptation to drink, as reported in the AASE questionnaire, was related to higher amygdala mGluR5 DVR.

Preclinical research implicates mGluR5 signaling in alcohol use disorder.^7–9^ Ethanol exposure can increase mGluR5 levels in the nucleus accumbens.^10,11,13,^ A complex pattern of findings suggests that intermittent alcohol exposure can transiently increase mGluR5 levels in the central nucleus of the amygdala.^10,12,^ Reversely, systemic administration of mGluR5 NAMs, at doses that occupy 50–80% of mGluR5, can suppress self-administration of ethanol without impairing self-administration of food.^7^ Direct intracranial injections of mGluR5 NAMs into the amygdala and nucleus accumbens reduce alcohol intake and the reinstatement of alcohol seeking in rodents.^11–16^

Clinical studies on mGluR5 in alcohol use disorder are scarce. A post-mortem investigation of brain samples found no alcoholism-associated alterations in mGluR1/5 density in the nucleus accumbens, putamen and caudate.^35^ An in vivo PET study using the mGluR5-selective radioligand 18F-FPEB reported reduced tracer binding in subjects with alcohol use disorder.^17^ Of note, approximately 69% of individuals with alcohol use disorder and none of the controls in that study were smokers.^17^ This is especially relevant, as we previously showed marked global reduction in mGLuR5 binding in smokers.^18,19,^ These global effects were particularly strong in brain regions that overlap with the anatomical distribution of the effects reported by Leurquin-Sterk et al.^17^

Based on our previous investigations on mGluR5 in humans, we carefully controlled for clinical variables that might confound our findings in the present investigation. Since we showed reduced mGluR5 DVR in depression in a previous study,^33^ we excluded patients with current major depressive episodes and other mood disorders from our present investigation. Moreover, given that smoking is associated with a global reduction in mGluR5, we also excluded smokers.^18,19,^ As rodent data suggest that antidepressants might increase mGluR5 levels,^36,37,^ we tested for a possible confounding effect in the present study. Amygdala mGluR5 DVR was lower in patients treated with antidepressants than in patients who did not receive antidepressants. Thus, our finding of higher amygdala mGluR5 DVR in patients, as compared to controls, cannot be explained by antidepressant medication.

Studies in rodents show that mGluR5 signaling is involved into the reinstatement of alcohol and nicotine self-administration.^7,8,^ In humans, we found that in ex-smokers mGluR5 DVR was related to the risk for relapse.^19^ Based on these results, we investigated whether mGluR5 DVR relates to the risk for relapse in alcohol use disorder. We did not find evidence that mGluR5 DVR was predictive of relapse in patients with alcohol use disorder.

We observed higher positive relation between mGluR5 DVR in the ACC and OFC in patients. This finding is particularly interesting in view of the fact that resting-state connectivity between the ACC and OFC, as measured with fMRI, is higher in alcoholics than in healthy controls.^23^ These converging results, from PET and fMRI, appear plausible from neuroanatomical point of view since both structures are structurally and functionally connected.^38,39,^ Moreover, these regions are critical for reward-related behavioral control and flexibility, both of which are impaired in addiction.^40–43^

In conclusion, our study provides clinical evidence for the implication of mGluR5 in the amygdala, OFC and ACC in alcohol use disorder.^3,4,^ Our findings strengthen the case for mGluR5-targeted pharmacological treatment of alcohol use disorder.^5^^,^^7–9^^,^^44^ mGluR5 NAMs have shown promising results in preclinical addiction models.^7^ Partial mGluR5 NAMs also show promising results and are currently in development.^45,46,^ The present study suggests that these pharmacological agents may be helpful in the treatment of alcohol use disorder.^5^

## Electronic supplementary material

Supplemental material
