# Supplementary material for: Metabotropic glutamate receptor 5 binding in male patients with alcohol use disorder
Source: Transl Psychiatry. 2018 Jan 10;8:17. doi: 10.1038/s41398-017-0066-6 (PMC5802584; doi:10.1038/s41398-017-0066-6)
Supplement: Supplementary file 1 — Supplemental material [file 41398_2017_66_MOESM1_ESM.doc]

**Metabotropic Glutamate Receptor 5 Binding in Male Patients with Alcohol Use Disorder**

**Running Title:** Metabotropic Glutamate Receptor 5 in Alcoholism

Funda Akkus, M.D., Yoan Mihov, Valerie Treyer, Ph.D., Simon M. Ametamey, Ph.D., Anass Johayem, Ph.D., Ph.D., Smeralda Senn., M.Sc., Susanne Rösner, Dr. hum. biol., Alfred Buck, M.D., Gregor Hasler, M.D.

**SUPPLEMENTARY INFORMATION**

Supplementary Table 1

| **Structure 1** | **Structure 2** | **r in Controls** | **r in Patients** | **r Difference** | **p (2-tailed)** |
| --- | --- | --- | --- | --- | --- |
| OFC Middle | Anterior cingulate | 0,1380 | 0,9106 | -0,7726 | 0,00055 |
| Straight gyrus | Anterior cingulate | 0,1667 | 0,9114 | -0,7447 | 0,00067 |
| OFC Posterior | Anterior cingulate | 0,1355 | 0,9052 | -0,7697 | 0,00069 |

Correlations between mGluR5 DVR in pairs of brain structures (***Structure 1*** and ***Structure 2***) that differed between patients and controls. Correlations are sorted according to the statistical significance of their difference between both groups. Brain regions are labelled according to the brain volumes implemented in PMOD (http://doc.pmod.com/pneuro/7674.htm). ***r in Controls / Patients*** indicates Pearson’s correlation coefficient for the respective group. ***r Difference*** indicates the numeric difference between the correlation coefficients in both groups **r_Controls_ - r_Patients_**. ***p-values*** refer to the significance of the correlation difference between patients and controls (Fisher’s r-to-z transformation, one-tailed, p < 0.001, uncorrected for multiple testing, as implemented in the R package “cocor”, version 1.1-3, in R, version 3.3.2, R Foundation for Statistical Computing, Vienna, Austria).

**Supplementary Figure 1**

Correlations between mGluR5 DVR in 36 regions, across the entire brain, in healthy controls. Brain regions are labeled on the left side and the top. Color heat represents the numeric value of the Pearson correlation between each pair of brain regions, as indicated in the color bar on the right: red indicates high positive correlations, whereas blue indicates high negative correlations.

**Supplementary Figure 2**

Correlations between mGluR5 DVR in 36 regions, across the entire brain, in patients recovering from alcohol use disorder. Conventions as in ***Supplementary Figure 1***.

**Supplementary Figure 3**


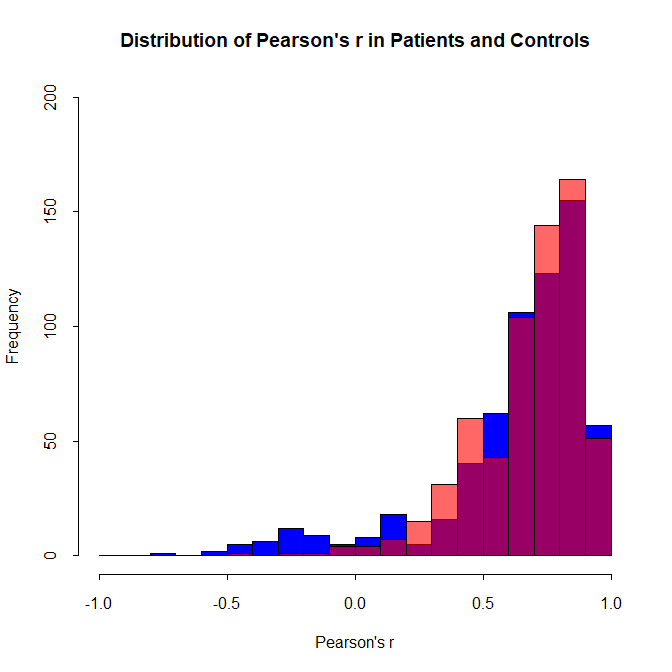


The distribution of Pearson’s r correlations of mGluR5 DVR among brain regions. Solid blue, in the background, represents the distribution of Pearson’s r in controls. The distribution of Pearson’s r in patients appears in the foreground in semitransparent red. Thus, overlapping areas appear purple, whereas non-overlapping areas are either light red (patients only) or solid blue (healthy controls only).

**Supplementary Figure 4**

We carried out a simulation with randomly generated data to systematically examine the validity of our correlation analyses[[1]](#footnote-2). We created 10,000 data sets each comprising two samples of 36 numeric vectors of fourteen values to simulate 10,000 pairs of groups (controls vs. cases) with 36 brain regions and fourteen subjects each. The fourteen values for each brain structure in each group and each data set were generated with the ‘rnorm’ function in R, based on a normal distribution with a mean = 0 and a standard deviation = 1. This resulted in 36*2*10,000 = 720,000 independent random vector generations. Following this, we repeated our original correlation analyses with each set of randomly generated data. We calculated 630 Pearson’s r correlation coefficients for each randomly generated data sample. We compared each of these 630 Pearson’s correlations between both samples of the same data set at the significance level used in our original analyses (p < 0.001, one-tailed). This resulted in 630*10,000 = 6,300,000 comparisons. To inspect the results, we plotted the percentage of randomly generated datasets conditioned on the number of significantly different correlation pairs they yielded (Supplementary Figure 4).

In 45.69% of the randomly generated data sets not a single pair of significantly different correlations between both samples was found. In 36.2% of these data sets only one correlation pair differed significantly. Two correlation pairs differed significantly in 13.56%, three correlation pairs in 3.79%, four correlation pairs in 0.63%, and five in 0.13% of the randomly generated data sets. In none of the randomly generated data sets more than five correlation pairs differed significantly. Thus, the fraction of data sets containing the same or a larger number of significantly different correlation pairs as in our original analyses (Supplementary Table S1), without further regard to the direction of these differences, was 3.79% + 0.63% + 0.13% = 4.55%. This fraction of false positives was within the commonly accepted alpha error-range of 5%.

**Supplementary Figure 5**

We carried out 10,000 two-tailed Welch’s tests, to compare the z-transformed correlations between both randomly generated samples in each data set, as with our original data (p < 0.05, two-tailed). To examine these results, we plotted the distribution p-values for 10,000 comparisons (Supplementary Figure 5).

We found that 5.31% of Welch’s tests comparing 630 z-transformed Pearson’s correlation coefficients between both samples yielded significant results. With non-transformed Pearson’s r-values the difference between both samples was significant in 5.34% of data sets. This fraction of false positives corresponds well to the accepted alpha error of 5%.

In conclusion, data simulation strengthened the validity of our correlation analyses. Randomly generated data yielded results that were similar to the findings in our original analyses in 5% of the simulated data sets. This rate of false positives corresponded to an alpha error-level of 5%.

1. The R simulation code can be obtained on request from author Y.M. (yoan.mihov@puk.unibe.ch). [↑](#footnote-ref-2)
